# *Streptomyces thermoautotrophicus* does not fix nitrogen

**DOI:** 10.1038/srep20086

**Published:** 2016-02-01

**Authors:** Drew MacKellar, Lucas Lieber, Jeffrey S. Norman, Anthony Bolger, Cory Tobin, James W. Murray, Mehtap Oksaksin, Roger L. Chang, Tyler J. Ford, Peter Q. Nguyen, Jimmy Woodward, Hugo R. Permingeat, Neel S. Joshi, Pamela A. Silver, Björn Usadel, Alfred W. Rutherford, Maren L. Friesen, Jürgen Prell

**Affiliations:** 1 Harvard Medical School, 200 Longwood Ave, Boston MA 02130; 2 Wyss Institute, 3 Blackfan Cir, Boston MA 02115; 3 Facultad de Ciencias Agrarias, Universidad Nacional de Rosario, Zavalla S2125ZAA, Santa Fe, Argentina; 4 Dept. Life Sciences, Imperial College, London, SW7 2AZ, UK; 5 Department of Plant Biology, Michigan State University, 612 Wilson Rd, East Lansing, MI USA 48824; 6 Institute for Biology I, BioSC, RWTH Aachen University, Worringer Weg 3, 52074 Aachen, Germany; 7 TheLab Inc, 1340 E. 6th Street Suite 603, Los Angeles, CA USA 90021

## Abstract

*Streptomyces thermoautotrophicus* UBT1 has been described as a moderately thermophilic chemolithoautotroph with a novel nitrogenase enzyme that is oxygen-insensitive. We have cultured the UBT1 strain, and have isolated two new strains (H1 and P1-2) of very similar phenotypic and genetic characters. These strains show minimal growth on ammonium-free media, and fail to incorporate isotopically labeled N_2_ gas into biomass in multiple independent assays. The *sdn* genes previously published as the putative nitrogenase of *S. thermoautotrophicus* have little similarity to anything found in draft genome sequences, published here, for strains H1 and UBT1, but share >99% nucleotide identity with genes from *Hydrogenibacillus schlegelii*, a draft genome for which is also presented here. *H. schlegelii* similarly lacks nitrogenase genes and is a non-diazotroph. We propose reclassification of the species containing strains UBT1, H1, and P1-2 as a non-Streptomycete, non-diazotrophic, facultative chemolithoautotroph and conclude that the existence of the previously proposed oxygen-tolerant nitrogenase is extremely unlikely.

Biological fixation of gaseous elemental nitrogen into ammonia is an important part of the global nitrogen cycle^1^. Several bacteria and archaea are known to carry out this process, either solely for their own biosynthetic needs^2^, or else as symbiotic partners with certain eukaryotes^3^. In many environments, bioavailable nitrogen is limiting for net primary productivity^4^, and this relevance for agriculture and ecology has ensured that the biology of nitrogen fixation has been an active area of research since its discovery in the 19^th^ century.

Bacteria synthesize ammonia from dinitrogen via a family of homologous nitrogenase enzymes, of which the molybdenum-iron (MoFe) nitrogenase is the most common^5^. The MoFe nitrogenase protein combines with the homodimeric nitrogenase reductase Fe-protein to form an (αβγ_2_)_2_ octameric enzyme complex^6^, encoded by the structural genes *nifHDK*. Each enzyme complex contains two iron-sulfur (4Fe:4S) clusters, two (8Fe:7S) P-clusters, and two (7Fe:Mo:9S:C:homocitrate) FeMo cofactor active sites. Several accessory proteins are required for the synthesis and insertion of these complex cofactors, and thus various subsets of some 16 different additional *nif* genes are present in diazotrophs^7^. A set of six *nif* genes, *nifHDKENB*, has been proposed as a diagnostic criterion for diazotrophy^8^. The MoFe nitrogenase has promiscuous activity, and is able to reduce a number of substrates besides dinitrogen^9^. Of particular interest is its ability to convert acetylene into ethylene, which is often used as a proxy assay for nitrogenase activity.

While the exact mechanism of nitrogenase function remains unknown^10^, the stoichiometry is thought to require at least 16 molecules of ATP, as well as six low-potential electrons from ferredoxin or flavodoxin, per molecule of dinitrogen reduced^11^, plus two more electrons to generate one molecule of hydrogen, an obligatory side product of nitrogenase function. In addition to this high energetic cost, nitrogenase activity is reversibly inhibited by hydrogen (H_2_) and carbon monoxide (CO)^12^; the former of which is generated by nitrogenase itself, and the latter generated by heme oxygenase in root nodules^13^, as well as being present in significant concentrations near sites of geothermal activity or combustion. More importantly, nitrogenase is rapidly and irreversibly damaged upon exposure to oxygen^14^, due to the effect of oxygen on the enzyme’s metal cofactors^15^, perhaps exposed to the solvent upon the cyclic dissociation of the nitrogenase and the nitrogenase reductase^16^. This sensitivity has led to a proliferation of evolutionary strategies to reduce oxygen tension near active nitrogenases^17^.

In addition to the MoFe nitrogenase, some bacteria possess alternative nitrogenases that substitute iron or vanadium for molybdenum in the active site^18^, presumably for use when Mo is scarce. These alternative nitrogenases introduce a δ subunit encoded by *vnfG* or *anfG*, altering the stoichiometry of the nitrogenase complex, but the structural *vnfHDK* and *anfHDK* genes are homologous to their *nif* counterparts, and the corresponding enzymes have even greater sensitivity to O_2_, as well as lower nitrogenase activity, and greater relative hydrogen production, than the MoFe enzyme.

A fourth, unrelated class of nitrogenase has been reported in the thermophilic carboxydotroph *Streptomyces thermoautotrophicus* UBT1^19^. This strain was isolated from the soil overlying burning charcoal piles, and was studied by members of the laboratory of Ortwin Meyer, at the Universität Bayreuth, Bayreuth, Germany. UBT1 was described as an obligate chemolithoautotroph, capable of growth on CO or CO_2_ and H_2_, but not on complex carbon sources^20^. Nitrogen fixation in this strain was found to be significantly different from that of other diazotrophs: it was not inhibited by H_2_, CO, or O_2_. Further, *nif* genes were not detected by Southern blot, and the strain was not found to reduce acetylene to ethylene, suggesting that this was the first natural diazotroph to lack a nitrogenase homologous to MoFe. Fractionation of protein lysate was used to identify structural components of the nitrogenase, and the N-termini of the proteins isolated showed no homology to known nitrogenase subunits^21^. The specific activities reported for this enzyme were unusually low in comparison to traditional nitrogenases. Most interesting of all, the enzyme was found to be insensitive to oxygen in cell lysates, and actually depended upon its presence for the generation of the superoxide anion (O_2_^−^), which was proposed to be used as an electron donor for the reduction of dinitrogen.

A catalytic mechanism was proposed whereby CODH (enzyme St3) transfers electrons from water to oxygen via the oxidation of CO, a manganese superoxide dismutase (Mn-SOD; enzyme St2) transfers electrons from the resulting superoxide anion to a molybdenum hydroxylase, likely another CODH homolog (St1), which in turn uses them to reduce dinitrogen. The CODH that generates superoxide was found to have no nitrogenase activity^21^. Degenerate oligonucleotides designed against the N-termini of the purified proteins have been used to identify the candidate nitrogenase genes, and they were found to be homologous to known carbon monoxide dehydrogenases (CODH; enzyme St1 encoded by *sdnMSL*) and a superoxide dismutase (enzyme St2 encoded by *sdnO*)^22^.

Stimulated by these early results, we have sequenced the genome of *S. thermoautotrophicus* UBT1, provided by the original isolator, D. Gadkari, in two independent laboratories. We have also isolated two novel strains of *S. thermoautotrophicus* with the described phenotypic characters: strain H1 from another burning charcoal pile, and strain P1-2 from soil near an active coal seam fire. We have sequenced the H1 strain and find an average of 95% identity between its coding sequences and those of UBT1. The genome of strain P1-2 was not sequenced. Both draft genome sequences for strain UBT1, as well as the draft genome for strain H1, lack known nitrogenase genes (*nif*, *vnf*, or *anf*). Further, all three draft genomes lack coding sequences with high identity to the published *sdn* genes; instead we note that the *sdn* genes previously published show high identity with genes in *Hydrogenibacillus schlegelii* DSM2000, a draft genome of which is also presented here. Finally, we find that the H1 and UBT1 genomes possess multiple loci encoding CODH enzymes, which is also the case in another organism previously reported to be a diazotroph, and whose published genome sequence similarly lacks *nif* genes: *Pseudonocardia dioxanivorans*. We accordingly included this strain in our characterization of putative novel nitrogen fixation activities.

The UBT1, H1, and P1-2 strains all grow on CO and H_2_ gas on mineral media, as well as on pyruvate, despite the previous description of UBT1 as an obligate chemolithoautotroph. Growth was not observed on other carbon sources tested. The H1, UBT1, P1-2, and *P. dioxanivorans* CB1190 strains sustain minimal growth on media lacking NH_4_, but do not incorporate heavy-isotope-labeled N_2_ gas (^15^N_2_) into biomass as measured in attempts at different sites. These results are inconsistent with nitrogen fixation, but instead suggest that this organism can incorporate ammonia and other combined nitrogen species at exceedingly low concentrations. We propose reclassifying *Streptomyces thermoautotrophicus* as a non-Streptomycete, non-diazotrophic, facultative chemolithautotroph.

## Results

### Strain UBT1 and two new strains grow largely as previously described

**Figure 1:**
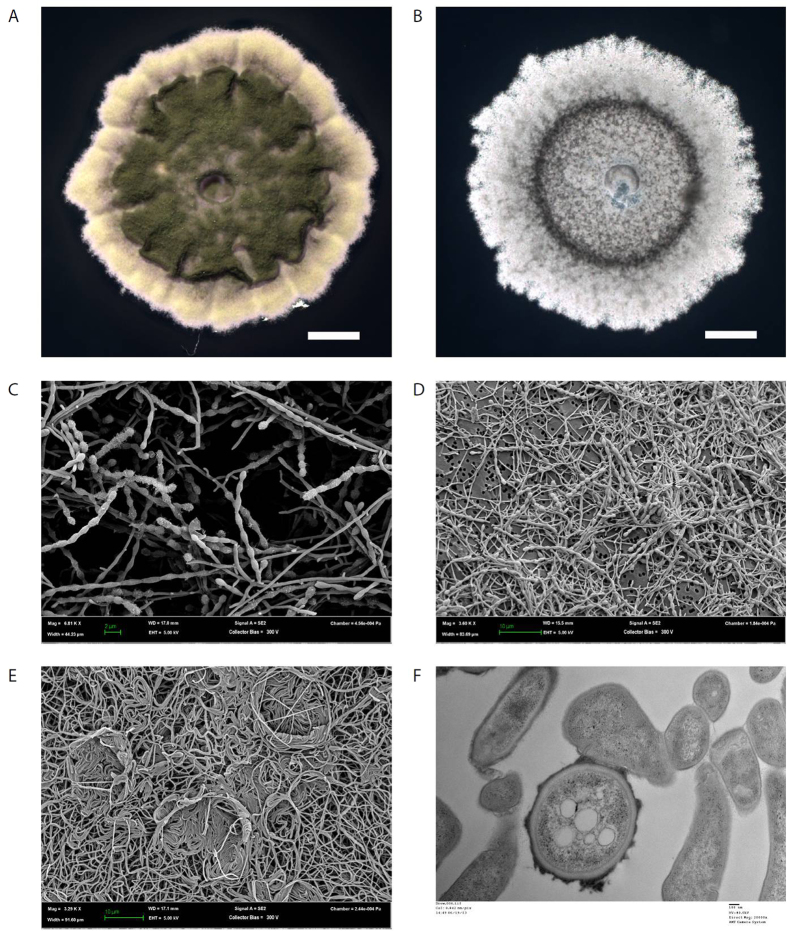
Morphology of *S. thermoautotrophicus*. On mineral media solidified with gellan gum, growth is more robust in the presence (**A**) than the absence (**B**) of 30 mM NH_4_Cl (scale bars are 200 μm in each image). Electron microscopy shows smooth, branching substrate mycelia, and hairy/rugose decorations on mature spores, which are arranged in straight, unbranched chains ((**C**), magnification is 6,810x). Spore formation is robust on media containing NH_4_Cl ((**D**), magnification is 3,600x), but when grown on media lacking NH_4_Cl the ratio of substrate to aerial mycelia is higher, and the hyphae assume varied morphologies ((**E**), magnification is 3,290x). In cross section, unknown vacuoles and other subcellular features appear, and may be more prominent in mature spores (lower center, identifiable by rugose exterior) than in other hyphae ((**F**), magnification is 20,000x).

The sources of the *Streptomyces thermoautotrophicus* strains used in this paper are summarized in Table 1. We found the morphology and general growth characteristics of all strains to be consistent with the previous description for strain UBT1^20^ (Fig. 1). Specifically, *Streptomyces thermoautotrophicus* grows rapidly on hydrogen, reaching its maximal extent of growth within three days of incubation at 60 °C on solid media in the presence of a 4:4:1:1 mix of N_2_:H_2_:CO_2_:O_2_. The strains grow more slowly on a 9:8:2:1 mix of CO:N_2_:O_2_:CO_2_, reaching maximal growth within four days of incubation at 60 °C. Limited germination could be observed on some carbon sources in the absence of H_2_ or CO, but the only carbon source found to sustain growth reproducibly was pyruvate. Growth in liquid was poor, either in suspension in pyruvate or as a pellicle in the presence of H_2_ or CO. Serial passage on mineral media solidified with gellan gum and in the presence of H_2_ or CO was robust, as was serial passage on plates containing pyruvate and NH_4_ (DSM260 media), suggesting that the initial assessment of this species as an obligate chemolithoautotroph was premature.

The strains did not grow above 80 °C or below 42 °C. H1 and UBT1 form a non-fragmenting, branched substrate mycelium and, after at least 24 hours of growth, produce aerial hyphae that septate into chains of grey-pigmented spores, the most mature form of which bear hairy to rugose surface decorations^23^. Substrate hyphae, aerial hyphae, and free spores are all highly hydrophobic, and remain buoyant atop aqueous solutions.

*S. thermoautotrophicus* strain H1 was isolated from soil obtained from a charcoal pile in Hasselfelde, Germany, by adding soil particles to liquid media under a hydrogen atmosphere, and transferring the resulting buoyant mycelia after 9 days of culture. The original liquid phase enrichment of *S. thermoautotrophicus* from soil retained spores that formed non-mycelial colonies, which grew well on complex media. 16S rRNA analysis of these colonies indicated that they were primarily related to thermophilic Firmicutes, including *Bacillus*, *Brevibacillus*, and *Geobacillus*. Interestingly, liquid phase growth of this initial consortium resulted in extensive pellicle growth, which was lost upon subsequent purification of *S. thermoautotrophicus* after serial passage and selection of single colonies on plates. Growth of the selected strain on plates remained robust, however.

*S. thermoautotrophicus* strain P1-2 was isolated from soil situated near an active coal seam fire in Centralia, PA, USA. Its nutritional requirements were identical to strain H1. PCR with universal RNA primers (27F and 1492R^24^) produced a single amplicon, Sanger sequencing of the amplicon indicated 99.3% identity with one of the 16S rRNA loci present in both genomes from the H1 and UBT1 strains (Supplementary Fig. 1).

### Genome sequences indicate a novel genus

**Figure 2:**
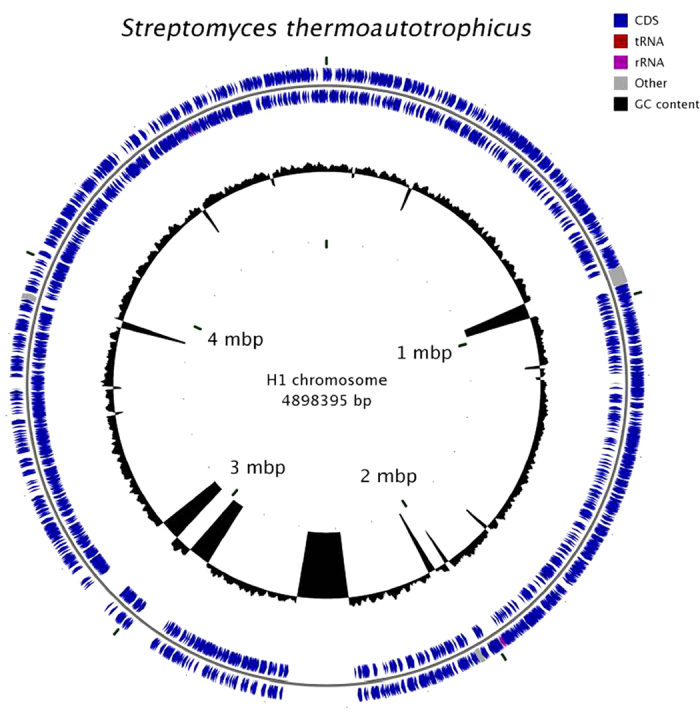
Genome map of *S. thermoautotrophicus* H1. 20 scaffolds from the SPAdes assembly were aligned to the optical map, and gaps in the chromosome were filled in with N’s manually. Six nodes did not align; the largest of which is a predicted plasmid. The two outer rings represent genes on the two strands of the chromosome. The inner ring shows the GC content, and prominent dips represent gaps in the assembled sequence; with length and position inferred from the optical map.

*Streptomyces thermoautotrophicus* strain UBT1 was grown and sequenced independently at two different facilities (Rosario, Argentina, and Aachen, Germany), and *S. thermoautotrophicus* strain H1 at a third (Boston, MA, USA; Fig. 2). Optical mapping data (OpGen, Gaithersburg, MD) were collected for strain H1, and indicated the presence of a single, circular chromosome of 5.25 Mbp. A combination of short reads (Illumina), scaffolding reads (Pacbio), and optical mapping consolidated genome assembly of strain H1 into a single scaffold 4.90 Mbp in length (of which 4.5 Mbp is sequenced data, and 0.4 Mbp is scaffolding Ns), containing an estimated 85% of the genome, with 5 additional contigs >1 kbp in length that could not be aligned to the map (and that could collectively account for another 1.3% of the genome), and one 48 kbp contig that is likely a plasmid, based on the presence of multiple genes encoding phage- and plasmid-related putative functions. The genome sequences of strain UBT1 are more fragmentary, but have similar lengths and gene content, including the possession of a 55 kbp plasmid (which shares no significant similarity with that of the putative H1 plasmid). The genome characteristics are compared to those of other Actinomycetes in Supplementary Table 1. Comparison of the H1 and UBT1 (Aachen) genomes by the Genome Blast Distance Phylogeny (GBDP) approach, as implemented with default settings and bootstrapping on a web server (http://ggdc.dsmz.de; date of access 21/06/2015)^25^ yielded a distance estimate of 0.0067, comparable to a DNA-DNA hybridization (DDH) value of 94.9%, predicting a 97.18% probability that DDH >70%, and thus that the strains belong to the same species.

The circularity and relatively modest size of the H1 genome are uncommon among Streptomycetes^26^, and accordingly we undertook analysis of the nearest relatives of these strains. The UBT1 (Aachen) genome assembly contains three small subunit ribosomal RNA genes, while the more fragmentary Rosario genome assembly contains two. The H1 strain genome assembly similarly has two. Each genome contains two different 16S gene sequences that share only 90% identity between them. Between the H1 and UBT1 strains, however, each 16S sequence in one strain matches the corresponding sequence from the other strain with 99% identity. The presence of significantly different ribosomal RNA operons within a single bacterial genome has been observed before, especially in thermophiles^27^. A phylogenetic tree comparing the 16S sequences shows that one of the 16S genes clusters most closely with *Thermobispora bispora* and *Acidothermus cellulolyticus* (Supplementary Figure 2a). The identities with either species are 90%. The other 16S sequence in either genome has various nearest neighbors among other actinomycetes, but shares less than 93% identity over 93% of its length with any of them, as determined by BLAST.

In the absence of conclusive evidence as to which of these divergent rRNA sequences is ancestral to the organism, and which may be the product of a more recent horizontal gene transfer or duplication event, we adopted the approach of concatenating 14 well-conserved proteins (13 ribosomal proteins and one phosphpatidate cytidylyltransferase) within the genome sequences of strains H1 and UBT1, and performing alignment and neighbor-joining analyses with similar sequences from all Actinobacteria with fully sequenced genomes that were available at the time of analysis^28^. The resulting tree, while possessing some nodes with low confidence based on the supporting bootstrap values, is largely in agreement with recent results that have followed the same approach^29^ (Supplementary Figure 2b). From this approach, it appears that H1 and UBT1 are most closely related to the clade that includes the genera *Acidothermus*, *Streptosporangium*, *Thermobifida*, *Thermobispora*, and *Thermomonospora*. By this analysis, these strains do not belong in the genus *Streptomyces*, and instead are in the class *Actinobacteria*, subclass *Actinobacteridae*, order *Actinomycetales*. The suborder, family, and genus are all undetermined, although nearby families include *Frankineae* (*Acidothermus*), *Streptosporangineae* (*Thermomonospora*, *Streptosporangium*, *Nocardiopsis*, *Thermobifida*), and *Pseudonocardineae* (*Thermobispora*).

### Genomes lack nitrogenases; possess multiple CODHs

No *nif* genes are present in any of the three draft genomes, other than a single gene annotated as “Nitrogen-fixing NifU, C-terminal:Rieske [2Fe-2S] region”. This single gene is located between a predicted uptake hydrogenase and factors for the maturation of that enzyme, which is homologous to similar clusters in other organisms. Proteins with C-terminal homology to NifU have predicted functions in metal cofactor synthesis, including in non-diazotrophs, and are not diagnostic for the presence of a functional nitrogenase^30^. The genome also contains no sequences with significant homology to *anf* or *vnf* genes for the alternative Fe- or V-nitrogenases.

**Figure 3:**
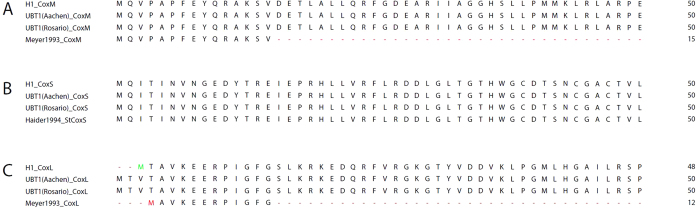
Gene sequences from the H1 and UBT1 genomes match closely those reported early in the cultivation of UBT1 by the Meyer group. Protein alignments from *S. thermoautotrophicus* CODH published by the Meyer group in 1993 versus corresponding sequences from the genomes of UBT1 and H1; CoxM (**A**), CoxS (**B**), and CoxL (**C**), respectively. For brevity, only the first 50 residues are shown for each alignment. N-terminal Edman degradation sequences for CoxM and CoxL are from^31^. The complete CoxS sequence was obtained from an unpublished dissertation from the Meyer laboratory; the full-length protein sequence possesses 100 percent identity with either H1 or UBT1 CoxS. Residues that are identical are black; residues that are similar to the consensus are colored green; all others are colored red.

The superoxide-dependent nitrogenase was previously identified to involve two molybdenum hydroxylases, St1 and St3, which were homologs of CODH. St1 was reported to be the putative dinitrogen reductase, and St3 a true CO dehydrogenase responsible for generation of the superoxide anion radical. Both were reported to possess the same heterotrimeric structure of typical CODH enzymes or other molybdenum hydroxylases^22^. The UBT1 genome contains four operons encoding predicted aerobic CODH, and the H1 genome contains three (Table 2). Two of the UBT1 operons contain large subunit (*coxL*) genes encoding the AYXCSFR motif characteristic of form I aerobic CODH^31^, and are located near *coxDEF* or *coxG* accessory genes necessary for the maturation of the functional CODH complex. The other two loci contain large subunits with alternative motifs that suggest categorization with form II CODH, or other molybdenum hydroxylases^32^. The medium subunits of all four loci have sequences consistent with the ability to bind flavin adenine dinucleotide (FAD) cofactor, despite the previous finding that St1 (encoded by *sdnM*) does not contain FAD^22^. One of the *coxMSL* loci, present in both the H1 and UBT1 genomes, shows high identity between the encoded proteins and sequences published by Ortwin Meyer in 1993^31^ (Fig. 3), suggesting that the UBT1 strain sequenced here is the same strain as reported on in that period.

The identity between the N-terminal protein sequences published for the St1 proteins with any of the UBT1 or H1 CODH sequences, however, is low (maximum identity between any H1 or UBT1 CODH protein and St1M, S, and L respectively is 53%, 50%, and 35%)^21^. Nucleotide sequences coding for the St1 subunits, based on sequencing clones from a library of DNA that hybridized with degenerate oligonucleotides derived from the N-terminal St1 sequences, have previously been determined^33^. These sequences have also recently been uploaded to the GenBank database (GI: 589823865, 589823867, and 589823869, for *sdnMSL*, respectively). The highest identity of any of the proteins encoded by these genes to any of the H1 or UBT1 sequences at the amino acid level is 50%, 63%, and 62%, for the proteins encoded by *sdnM*, *S*, and *L*, respectively (Table 2). These proteins in our *S. thermoautotrophicus* genomes are all annotated as CoxM, S, and L proteins.

The St2 protein was previously identified as a manganese-dependent superoxide dismutase (Mn-SOD), encoded by the *sdnO* gene. Similarly to St1, a nucleotide sequence for the entire *sdnO* gene had been recorded^33^, and has recently been uploaded to GenBank (GI:588295007). The UBT1 and H1 genomes each contain one predicted Fe/Mn-SOD; identity with the St2 protein sequence encoded by the reported *sdnO* gene sequence is 38% at the amino acid level (Table 2).

### Superoxide-Dependent Nitrogenase genes match *Hydrogenibacillus schlegelii*

The proteins encoded by *sdnMSL* and *sdnO* have higher identities, however, with proteins from other strains which have partial sequences within the GenBank database. A BLAST search revealed that the closest match for *sdnL* was to a partial coxL gene sequence (GI:38679249) in *Hydrogenibacillus schlegelii* (formerly *Bacillus schlegelii*), for which no full genome sequence was available. In order to investigate the relationship between the *sdn* sequences and *H. schlegelii*, we undertook sequencing the genome of that organism as well, and report here a draft sequence for the genome of *H. schlegelii* strain DSM2000. This strain is a known chemolithoautotroph, with similar optimum temperature for growth as *S. thermoautotrophicus* and similar capacity to grow on CO or H_2_. Accordingly, the draft genome contains genes encoding ribulose bisphosphate carboxylase/oxygenase (RuBisCo), phosphoribulokinase (PRK), CODH and uptake hydrogenase. *H. schlegelii* has not been described as a diazotroph. Its genome lacks *nif*/*anf*/*vnf* nitrogenase genes, and we found that the strain failed to grow on media lacking NH_4_Cl, with no visible turbidity in cultures incubated for up to one month, either when grown chemolithotrophically on H_2_/CO_2_, or when grown heterotrophically on pyruvate.

The DSM2000 genome contains a single locus encoding a putative molybdenum CODH operon (TR75_12445-55) with >99% identity with the *sdn* operon at the nucleotide level. Translations of the open reading frames (ORFs) within also demonstrated >99% identity to the St proteins (Table 2). It was further determined that a Mn-SOD within the *H. schlegelii* genome (TR75_10445), bore 100% identity at the amino acid level to the St2 protein (Table 2). Extensive overlap was also found at the nucleotide level between the DSM2000 genome sequence and the region around the coding sequence for *sdnO* (99.62% identity, over 7.2 kbp), previously sequenced by the Meyer group.

### Strains grow on media lacking NH_4_Cl, but fail to incorporate significant ^15^N_2_ into biomass

All *S. thermoautotrophicus* strains tested were capable of continuous culture on mineral media lacking added NH_4_^+^ but peculiarities regarding their growth were apparent. Specifically, growth was far more robust in the presence of 30 mM NH_4_^+^. Furthermore, growth was only supported on plates solidified with 0.5–1.0% gellan gum, or on agar fortified with charcoal or bio-char. A CHN analysis on the gellan gum used returned a nitrogen content of 0.095%. Growth on mineral media solidified with Noble agar was not observed. In addition, growth on plates containing either gellan gum or agar plus bio-char was greatly enhanced by the presence of plates containing 30 mM NH_4_^+^ within the same atmosphere, presumably due to the former cross-feeding on NH_3_ volatilizing from the latter.

The high number of CODH enzymes encoded by the *S. thermoautotrophicus* genomes and the purported identity of the St1 nitrogenase as a modified CODH prompted us to examine other published genomes containing high numbers of such enzymes. We identified *Pseudonocardia dioxanivorans* CB1190 as another strain whose sequenced genome encodes multiple molybdenum hydroxylase enzymes (10 operons, two of which are putative type I CODH enzymes^34^), and which has been reported to be a diazotroph^35^. The strain CB1190 similarly lacks *nif*, *anf*, and *vnf* genes, yet reportedly displayed a similar ability to grow on media lacking NH_4_^+^ as an additive. This strain was accordingly included in subsequent experiments to test for diazotrophy using isotope labeling.

In order to determine whether the growth of our strains was independent of exogenous combined nitrogen, we undertook labeled nitrogen assays at four independent laboratories (Table 3). Plates were inoculated with spores of the strains indicated and incubated in the presence of acid-washed ^15^N_2_ gas. After 3–5 days, biomass was scraped from plates and subjected to isotope-ratio mass spectrometry (IRMS). At three sites with thorough ^15^N_2_ gas washing, no enrichment of ^15^N was detected in biomass relative to plates supplemented with NH_4_Cl. At one site, a slight enrichment of ^15^N in biomass was observed. We note, however, that the ^15^N_2_ gas used in this experiment (Aldrich catalog #364584, Lot#SZ1670V) was found in a recent report^36^ to contain significant amounts of contaminating ^15^NH_4_^+^ and ^15^NO_3_^−^/NO_2_^−^. The small amount of ^15^N detected in the biomass of our samples, is much less than the amount that would be present in unwashed gas (Supplementary Fig. 3); thus it is possible that not all of this contaminating fixed ^15^N was captured by washing for 30 minutes in 5% H_2_SO_4_. Fixation was also not detected in *P. dioxanivorans* CB1190.

## Discussion

We conclude that there is no evidence for, and substantial evidence against, the existence of an oxygen-tolerant nitrogease as previously described in *Streptomyces thermoautotrophicus*. Our major empirical lines of evidence are both genomic and phenotypic. First, using genome sequencing, we failed to find either canonical or the proposed novel nitrogenase enzymes in our *S. thermoautotrophicus* isolates. Rather, sequences of the proposed enzymes are present in another thermophilic, non-diazotrophic bacterium. Second, extensive multi-site fixation experiments using ^15^N_2_ gas failed to demonstrate incorporation into biomass. Growth experiments suggest that *S. thermoautotrophicus* strains are highly effective nitrogen scavengers, but not nitrogen fixers.

Finally, we point out some other inconsistencies in the original characterization of the proposed oxygen-tolerant nitrogenase system. Apart from the superoxide dependent nitrogenase, no known aerobic reduction of nitrogen to ammonia is known. An unprecedented mechanism in the scheme is the use of superoxide as an electron donor for a biologically productive reaction. No other productive biological use of superoxide is known, although many cells produce superoxide as a toxin. Usually superoxide is a toxic byproduct of cellular metabolism. With a standard potential of −330 mV^37^ it is comparable to the NADH or NADPH potential of −320 mV, but much more toxic due to its reactivity. The Fe protein of the MoFe nitrogenase has an apparent midpoint potential of −470 mV^38^, and the physiological electron donor is ferredoxin or flavodoxin. Therefore it seems unlikely that superoxide is sufficiently reducing to drive the nitrogenase reaction. In addition, none of the putative superoxide dependent nitrogenase system proteins have a plausible ATPase domain identified, even though ATP hydrolysis was required for the activity. In the original work, labeled nitrogen was not used during the purification of the nitrogenase, instead an ammonia production assay^21^ was used, based on the reaction of ammonia with sodium phenate and hypochlorite^39^. This assay is very sensitive, but is known to have a high background^40^, which may have contributed to a misidentification of nitrogenase activity.

There is absolutely no genomic evidence that *S. thermoautotrophicus* is diazotrophic. No MoFe, VFe, or Fe-only nitrogenase genes are present in the draft genome sequences presented here. The UBT1 strain was previously found to lack *nif* genes by dot blotting^19^, and its putative oxygen-tolerant nitrogenase was characterized by protein lysate fractionation and assays for ammonia production *in vitro*^21^. The identities of the three protein complexes of the proposed oxygen-tolerant nitrogenase were previously found to be two variants of molybdenum hydroxylases related to CODH, and an SOD-like protein^21^,^22^. However, N-terminal sequences of the proposed heterotrimeric St1 nitrogenase, and of the homodimeric St2 nitrogenase reductase, as well as the sequences of their respective genes *sdnMSL* and *sdnO*, do not match any sequences present in the draft genomes of strain UBT1 or the novel isolate H1^21^,^22^.

The genes for the proposed oxygen-tolerant nitrogenase are in fact present in the genome of another thermophilic non-diazotrophic bacterium. The sequences for *sdnMSL* and *sdnO* match with identities between 99.1% and 100% at the nucleotide level the *coxMSL* and *sod* gene sequences from *Hydrogenibacillus schlegelii DSM2000*, an isolate closely related to *H. schlegelii DSM9132*, which was studied by the Meyer group in the same period as *S. thermoautotrophicus*^41^,^42^,^43^. *S. thermoautotrophicus* and *H. schlegelii* overlap closely in their nutritional requirements and optimal growth temperature, and contamination of cultures of *S. thermoautotrophicus* is therefore a possibility. If the Meyer group did contaminate their liquid culture of *S. thermoautotrophicus* with *H. schlegelii*, these are the sequence data we would observe. We conclude that the St1 and St2 proteins are in fact the aerobic CODH and superoxide dismutase proteins from a strain of *Hydrogenibacillus schlegelii.*

In addition to the absence of the putative oxygen-tolerant nitrogenase genes, our extensive phenotypic characterization does not support the claim the *S. thermoautotrophicus* is diazotrophic. The strain of *S. thermoautotrophicus* we have received from D. Gadkari represent the closest known link to that which was claimed to be diazotrophic in 1992. Independent samples of that strain were cultured by different personnel at two independent sites, found to grow poorly on media lacking NH_4_Cl, grew reproducibly only on media containing additives that may contribute combined nitrogen, and did not incorporate ^15^N_2_ into biomass. We fail to see convincing evidence for diazotrophy in this strain. In addition, strain H1, isolated from a burning charcoal pile using procedures detailed for the isolation of UBT1 by the original authors^20^, and strain P1-2, isolated from soil overlying a coal seam fire, share morphology and physiology with UBT1 that are indistinguishable by any test performed thus far. They qualify as members of the same species as UBT1, according to whole-genome prediction of DNA-DNA hybridization and 16S rRNA sequence identity for strains H1 and P1-2, respectively. While there is no guarantee that independent isolates of a particular species of bacteria will share identical metabolic capabilities, it is striking that the H1 and P1-2 strains, which were not cultivated for prolonged periods in the laboratory before being subjected to ^15^N_2_ incorporation assays, show a similar capacity for minimal but reproducible growth on media lacking added NH_4_Cl, yet also fail to show significant incorporation of acid-washed, labeled dinitrogen into biomass.

Similar to our results in *S. thermoautotrophicus*, the abundance of CODH paralogs in the genome of *P. dioxanivorans* failed to predict functional diazotrophy in that strain, as measured by ^15^N_2_ assays. Subsequent dialogue with the authors responsible for the isolation and initial characterization of the *P. dioxanivorans* species confirmed that, contrary to early reports, the sequenced strain lacks known nitrogenase genes, and that the strain now available is not diazotrophic (L. Alvarez-Cohen, personal communication).

Other researchers have identified bacteria capable of growth on trace environmental sources of combined nitrogen^44^,^45^, and previous claims of diazotrophy in various strains have subsequently been found to be premature^46^,^47^. A high-affinity pathway for assimilation of scarce combined nitrogen may prove a viable alternative to the maintenance of a complex pathway for nitrogen fixation when ammonia and nitrate are scarce. Efficient scavenging of combined nitrogen is certainly a more parsimonious explanation for our data than a novel nitrogenase that is orthogonal to all known systems and insensitive to oxygen.

While it remains possible that novel nitrogenases will be uncovered as the resources for querying the genomes of unculturable bacteria advance, any such enzymes must be supported by sensitive functional assays in order to verify their activities. Among such assays, measuring the incorporation of isotopically-labeled dinitrogen gas into biomass by mass spectrometry remains the most sensitive and specific^48^,^49^. The high sensitivity of isotope-ratio mass spectrometry, however, can also be a liability, as when the input reagent may be contaminated with fixed ^15^N, a fact that may have contributed to the confusion regarding the status of UBT1 and other strains as putative diazotrophs.

We cannot rule out the possibility that the strains used in the previous publications cited possessed a novel nitrogenase, which was subsequently lost upon prolonged cultivation in the laboratory. Any such enzyme, however, would likely not be constituted of subunits with the sequences previously published, as their presence in *H. schlegelii* does not correspond with the ability to fix nitrogen, nor does their absence in UBT1 limit its ability to grow on media containing no added NH_4_Cl. Should a viable sample of diazotrophic UBT1 be recovered, we welcome the chance to characterize it further. However, given the evidence in hand we conclude that the existence of the proposed oxygen-tolerant nitrogenase system is extremely unlikely.

Regarding the reassignment of the phylogeny of this species: the identity between the 16S rRNA sequences in the H1, UBT1, and P1-2 strains is sufficiently high to warrant their classification as members of a single species. When compared to other Actinobacteria, however, identity falls below the commonly suggested guidelines for placing these strains within a known genus^50^. More sensitive protein sequence alignments support this divergence, and suggest an early-branching member of the order Streptosporangiales. It is unclear at the current level of resolution available from full genome sequences whether these strains best fit as members of the family Nocardiopsaceae, Thermomonosporaceae, or a new family. In any case, the level of divergence from categorized species is sufficient to recommend that *Streptomyces thermoautotrophicus* be reassigned to a new genus, and described as a non-diazotroph on the basis of this work.

The UBT1 (Rosario), H1, and P1-2 strains have been deposited in the Leibniz-Institut DSMZ (Deusche Sammlung von Mikroorganismen und Zellkulturen GmbH) culture collection, with catalogue numbers DSM 100163, DSM 100164, DSM 100422 respectively.

## Methods

### Strain isolation and cultivation

*S. thermoautotrophicus strain* UBT1 was kindly provided by the original author Dilip Gadkari, Bayreuth, Germany to research groups at the Universidad Nacional de Rosario, Argentina, and the RWTH Aachen, Germany. At the Universidad Nacional de Rosario, *S. thermoautotrophicus* strain UBT1 was grown in DSMZ Medium 811, in a defined atmosphere of 60% CO, 20% CO_2_, 20% air. Plates were grown on 1.5% agar supplemented with 0.5% activated charcoal. At the RWTH Aachen, *S. thermoautotrophicus* strain UBT1 was grown in NFIX mineral media supplemented with Drews elements (in μg/L: Na-Fe-EDTA 800, MnCl_2_ 10, CoCl_2_ 2, CuSO_4_ 1, ZnCl_2_ 2, LiCl 0.5, SnCl_2_*2H_2_O 0.5, H_3_BO_3_ 1, KBr 2, KI 2, BaCl_2_ 0.5, pH 6.0) in a defined atmosphere of 45% CO, 5% CO_2_, and 50% air.

*S. thermoautotrophicus* strain H1 was isolated from soil taken from a charcoal pile in Hasselfelde, Germany. Soil particles were immersed in mineral media^20^ and incubated in the presence of H_2_ and CO_2_ at 60° C. Pellicles emerged after several days, and were transferred and enriched on plates of mineral media plus 0.5% gellan gum (Sigma) and 0.075% MgCl_2_*6H_2_O in a defined atmosphere of 40% H_2_, 10% CO_2_, and 50% air, or else in 45% CO, 5% CO_2_, and 50% air^51^.

*S. thermoautotrophicus* strain P1-2 was isolated from soil located above an underground fire where a coal seam has been burning for several decades (Centralia, PA)^52^. The soil temperature *in situ* at the time of collection was 60 °C, and the collection site was immediately adjacent to steam issuing from a fissure. Isolation proceeded as for the H1 strain, except that isolation was conducted at an independent facility, liquid enrichment was in DSMZ261 mineral media^53^, and plates were solidified with 1% gellan gum and 0.15% MgCl_2_.

*Pseudonocardia dioxanivorans* CB1190 was grown on ATCC medium 196^35^ for routine maintenance, and was grown on NFIX mineral media plus glucose and gellan gum for labeled nitrogen assays. *Azotobacter vinelandii* strains DJ and DJ100 (Δ*nifD*)^54^ were grown on Burk’s medium^55^ with or without 30 mM NH_4_Cl for maintenance and labeled nitrogen assays. *P. dioxanivorans* and *A. vinelandii* were grown in air at 30 °C. *Hydrogenibacillus schlegelii* DSM2000 was grown on DSMZ Medium 260 or 261^56^, for heterotrophic or chemolithotrophic growth, respectively. To test for diazotrophy in this strain, variants of these media to omit NH_4_Cl were also prepared. *H. schlegelii* was grown at 55 °C in either air or an atmosphere of 45% H_2_, 10% CO_2_, and 45% air.

### Labeled nitrogen assay

In all experiments, separate canisters of ^15^N_2_ gas were purchased from Aldrich chemical (St. Louis, MO). The gas was acid-washed prior to introduction to the culture vessels: ^15^N_2_ gas was retrieved from the canister and injected into a Schott bottle filled with water and sealed with a rubber septum. Water was withdrawn upon introduction of the gas to equalize pressure and allow continued introduction of the gas until approximately half the volume of the bottle was occupied by the gas. Then concentrated H_2_SO_4_ was introduced to bring the concentration of H_2_SO_4_ in the water within the bottle to 5%. The gas was incubated for the indicated period of time with agitation before being extracted and introduced to the culture chamber.

At Harvard Medical School: ^15^N_2_ gas was acid-washed for 30 minutes. Plates of NFIX mineral media possessing or lacking 1.5 g/L (28 mM) NH_4_Cl and solidified with 0.5% gellan gum were inoculated with *S. thermoautotrophicus* H1 or UBT1 and incubated in the presence or absence of 2.5% ^15^N_2_ for 3 days. Then biomass was collected, dried at 80 °C for 12 hours, and analyzed at the MBL Stable Isotope Laboratory (Woods Hole, MA) on a Europa 20-20 CF isotope ratio mass spectrometer equipped with a Europa ANCA-SL element analyzer. *A. vinelandii* was grown in suspension in Burk’s medium possessing or lacking 1.5 g/L NH_4_Cl, with stirring, at 30 °C for 3 days.

At the RWTH Aachen: ^15^N_2_ gas was acid-washed for one hour. Plates of NFIX mineral media lacking 1.5 g/L (28 mM) NH_4_Cl and solidified with 1.0% gellan gum were inoculated with *S. thermoautotrophicus* H1 or UBT1 and incubated for five days in the presence of 10% ^15^N_2_ gas. Then biomass was collected, dried at 80 °C overnight, and sent for analysis to ISO-analytical, Crewe, UK.

At Universidad Nacional de Rosario: ^15^N_2_ gas was acid-washed for 30 minutes. Plates of NFIX mineral media possessing or lacking 1.5 g/L (28 mM) NH_4_Cl, fortified with 0.5% activated charcoal, and solidified with 1.5% agar were inoculated with *S. thermoautotrophicus* H1 or UBT1 and incubated at 60 °C for 5 days in desiccators in the presence or absence of 1.5% ^15^N_2_. *A. vinelandii* DJ and DJ33^57^ (*nifD*^−^ *nifK*^−^)were grown in Burk’s medium with (DJ33) or without (DJ) 1 g/L NH4 Cl for 3 days at 28 C in desiccators in the presence or absence of 0.75% ^15^N_2_. Then biomass was collected, samples were dried at 60 °C for 24 hours and analyzed at the MBL Stable Isotope Laboratory (Woods Hole, MA) on a Europa 20-20 CF isotope ratio mass spectrometer equipped with a Europa ANCA-SL element analyzer.

At Michigan State University: ^15^N_2_ gas was acid-washed for 24 hours. Plates of NFIX mineral media possessing or lacking 1.5 g/L (28 mM) NH_4_Cl were solidified with 1% gellan gum with NH_4_Cl and 6 plates without NH_4_Cl were each inoculated with UBT1, H1, and P1-2 strains. These plates were incubated for 3 days in 40% H_2_, 10% CO_2_, and 50% air atmospheres with 2% of the atmosphere replaced with acid-washed ^15^N_2_. We also incubated plates of *Azotobacter vinelandii* (wild type) and *A. vinelandii* (*nifD-* mutant), which cannot fix nitrogen in Mo-containing media, as positive and negative controls, respectively. Controls were incubated on mineral media with 1% glucose either with (*A. vinelandii nifD*^*−*^) or without (*A. vinelandii* WT) NH_4_Cl in the presence of an air atmosphere with ~2% of the atmosphere replaced with acid-washed ^15^N_2_. After incubation, growth was scraped from plates and washed in Phosphate buffered saline to remove potential contaminating nitrogen. Two NH_4_-free plates worth of biomass were pooled into single samples at this point to compensate for poor growth of H1, UBT1, and P1-2 strains on these media. Biomass was dried at 60 °C for 24 hours, then analyzed for ^15^N content on a GV instruments Isoprime Mass Spectrometer interfaced with a EuroVector Elemental Analyzer 3000 Series.

### Sequencing and bioinformatic analyses

At Universidad Nacional de Rosario: genomic DNA from *S. thermoautotrophicus* UBT1 (Rosario), was extracted with cetyltrimethylammonium bromide^58^. DNA was fragmented by nebulization, ends repaired, adaptors ligated, and small fragments removed following GS-FLX Titanium library preparation. Libraries were sequenced with single-end FLX 454. The reads were assembled with Newbler v.2.5.3^59^, and the genome annotated by RAST.

At the RWTH Aachen: genomic DNA from *S. thermoautotrophicus* UBT1 (Aachen), was prepared with a NucleopSpin Tissue kit (Macherey-Nagel, Germany). DNA was fragmented using a Biorupter Pico (Diagenode, Belgium), and libraries were prepared with the TruSeq kit. Libraries were sequenced with the 250PE reagent kit on a MiSeq (Illumina). PacBio reads were generated by GATC (Germany). Illumina reads were trimmed for quality with Trimmomatic (V0.32)^60^, and Illumina and PacBio reads were assembled with SPAdes version 2.5.1^61^. The resulting assembly was annotated with RAST.

At Harvard Medical School: genomic DNA from *S. thermoautotrophicus* strain H1 was isolated with the DNeasy blood and tissue kit (Qiagen). Quality was verified with Bioanalyzer chips (Agilent). DNA was fragmented by adaptive focused acoustics (Covaris) to 400 bp or 3 kb, and libraries prepared with the TruSeq kit. Libraries were sequenced with 250 PE reagent kits on a MiSeq (Illumina). PacBio reads were generated with the SMRTbell Template Prep Kit. Reads were trimmed for quality with Trimmomatic^61^, and assembled with SPAdes 3.1^61^. The resulting assembly was annotated with RAST^28^, augmented with a bidirectional BLAST^62^ search against all bacterial protein sequences from the UniProt database^63^ to increase the number of genes with a putative function.

At Michigan State University: genomic DNA from *H. schlegelii* DSM2000 was obtained directly from DSMZ, and was also extracted from *H. schlegelii* DSM2000 grown in Na-pyruvate mineral media (DSM 260: 4.5 g/L Na_2_HPO_4_, 1.5 g/L KH_2_PO_4_, 0.01 g/L MnSO_4_*7H_2_O, 0.2 g/L MgSO_4_*7H_2_O, 0.01 g/L CaCl_2_*2H_2_O, 0.005g/L Ferric citrate, 1.0 g/L NH_4_Cl,1.50 g/L Na-pyruvate, and 3.0 mL DSM trace element solution 6) without shaking at 60 °C. Cells were pelleted, incubated for 30 minutes at 55 °C with 280 ul Tissue Cell Lysis solution (Epicentre) and with 20 ul Proteinase K (Roche). 200 ul MPC protein precipitation solution was then added, the supernatant was transferred to isopropanol, and washed with 70% ethanol. Libraries were prepared for sequencing from both sources of DNA. Genomic DNA was sheared on a Covaris S2 ultrasonicator using the manufacturer’s recommended parameters for shearing to 500 bp. Sheared DNA was end repaired, A-tailed, and Illumina adapters were ligated using enzymatic kits from New England Biolabs^64^. Ligation products were amplified for 15 PCR cycles with Phusion polymerase (NEB) and size-selected on a 2% agarose gel. Finished libraries were pooled and sequenced on an Illumina MiSeq 150 bp paired sequencing run. Quality-filtered reads were assembled with the A5 pipeline^65^, and the resulting assembly was annotated with RAST.

The CoxMSL operon sequence from *H. schlegelii* DSM2000 was completed using the polymerase chain reaction (PCR). A DreamTaq kit (Thermo) was used to amplify for 35 cycles, and the amplicon was gel-purified and sequenced on an ABI 3730XL with BigDye chemistry. A quality-trimmed Sanger read was used to merge the two scaffolds in Phrap^64^,^66^.

Phylogenetic analyses were performed on 16S rRNA genes by downloading sequences from the NCBI, RefSeq Targeted Loci Project (revised Nov. 2010 at http://www.ncbi.nlm.nih.gov/genomes/static/refseqtarget.html; date of access 23/08/2015). 352 sequences representing Actinobacteria with sequenced genomes available were retrieved, as well as *B. subtilis* as an outgroup. These were aligned to the 16S sequences from the *S. thermoautotrophicus* genomes using Clustal Omega version 1.2.1^67^ with default parameters and 30 iterations of sequence input order. The resulting alignment was submitted to the Gblocks server^68^, and processed with the default settings. The consolidated alignment was used to form a distance matrix and a neighbor-joining tree with 100 bootstrap replicates, which were then used to form a consensus tree; these operations were carried out with PHYLIP version 3.695^69^. The final tree was edited in MEGA6^70^: for the condensed tree, branches that were monophyletic according to their current classification in NCBI Taxonomy were collapsed up to the family level.

Phylogenetic analyses were performed on protein sequences by identifying highly conserved sequences as previously described^28^. The subset of these represented in both the H1 and UBT1 genomes, including 14 ribosomal proteins and a phosphatidate cytidylyltransferase, were identified. Subsequently all Actinobacterial sequences, as well as *B. subtilis* as an outgroup, of the corresponding protein families were downloaded from the PFAM server^71^. These sequences were combined with the *S. thermoautotrophicus* proteins, and the proteins from each family were concatenated by species to form a single extended protein sequence representing a single isolate. The resulting table contained 431 strains, including H1 and UBT1. These were aligned in Clustal Omega with 30 iterations of input order, the resulting alignment trimmed with Gblocks, the trimmed alignment used to form a distance matrix and neighbor joining tree with 100 bootstrap replicates, and the consensus tree all generated with PHYLIP as described above. The final tree was edited in Mega to collapse monophyletic branches to the family level, excepting the immediate neighbors of H1 and UBT1.

## Additional Information

**How to cite this article**: MacKellar, D. *et al.* *Streptomyces thermoautotrophicus* does not fix nitrogen. *Sci. Rep.* **6**, 20086; doi: 10.1038/srep20086 (2016).

## Supplementary Material

Supplementary Figures 1 to 3

## Figures and Tables

**Table 1:** Sources of *S. thermoautotrophicus* strains used in this paper.

Strain	University	Isolation Site	Year of Isolation	Environment	Citation	Isolated By
UBT-1	Aachen	Plecht, Bavaria	1990	Charcoal pile soil	Gadkari 1990	D. Gadkari
UBT-1	Rosario	Plecht, Bavaria	1990	Charcoal pile soil	Gadkari 1990	D. Gadkari
H1	Boston	Hasselfelde, Saxony	2012	Charcoal pile soil	This work	C. Tobin
P1-2	East Lansing	Centralia, PA	2013	Coal seam fire soil	This work	J. Norman

**Table 2:** Predicted carbon monoxide dehydrogenase genes in strains H1 and UBT1, and *H. schlegelii*, compared to *sdn* genes.

Putative Function	H1	UBT1 Aachen	UBT1 Rosario	H. schlegelii	Hofmann-Findeklee 2000	% identity (boxed pair)	% identity (UBT1 Aachen & analogous Sdn protein)
CoxM	LI90_1082	TH66_09435	TR74_17685	—	—	100	44
CoxS	LI90_1083	TH66_09430	TR74_17680	—	—	100	61
CoxL	LI90_1084	TH66_09425	TR74_17675	—	—	100	62
CoxL	LI90_1777	TH66_03290	TR74_06615	—	—	100	39
CoxM	LI90_1775	TH66_03330	TR74_06625	—	—	99	50
CoxS	LI90_1776	TH66_03295	TR74_06620	—	—	100	35
CoxM	LI90_1945	TH66_19340	TR74_24565	—	—	100	48
CoxS	LI90_1944	TH66_19345	TR74_24560	—	—	100	30
CoxL	LI90_1946	TH66_19350	TR74_24570	—	—	100	35
CoxM	—	TH66_01980	TR74_16700	—	—	100	48
CoxS	—	TH66_01975	TR74_16695	—	—	100	63
CoxL*	—	TH66_01970	TR74_16690	—	—	100	59
CoxM	—	—	—	TR75_12455	SdnM	100	—
CoxS	—	—	—	TR75_12450	SdnS	99	—
CoxL	—	—	—	TR75_12445	SdnL	99	—
Fe/Mn SOD	LI90_840	TH66_04480	TR74_15150	—	—	100	38
Fe/Mn SOD	—	—	—	TR75_10445	SdnO	100	—

^*^mis-annotated as pseudogene; start codon is 21 bp 5′ to feature’s start.

**Table 3:** Results of growth of selected strains in the presence of ^15^N_2_ gas.

Strain	Site	Nutrition	^15^N_2_ (% of available N_2_)	Atom% 15N	n
*A. vinelandii* WT	Boston	Sucrose	2.5	2.35 ± 0.208	3
*A. vinelandii nifD*-	Boston	Sucrose/NH4	2.5	0.37 ± 0.004	3
H1	Boston	H2/CO2	2.5	0.43 ± 0.013	3
H1	Boston	CO	2.5	0.36 ± 0.018	3
UBT1	Boston	H2/CO2	2.5	0.43 ± 0.003	3
UBT1	Boston	CO	2.5	0.40 ± 0.012	3
H1	Boston	H2/CO2/NH4	2.5	0.36 ± 0.000	3
UBT1	Aachen	CO	10	0.37 ± 0.002	7
H1	Aachen	CO	10	0.37 ± 0.000	3
*A. vinelandii* WT	Rosario	Sucrose	0.75	0.79 ± 0.002	3
*A. vinelandii nifD*- nifK-	Rosario	Sucrose/NH4	0.75	0.37 ± 0.001	3
H1	Rosario	CO	1.5	0.36 ± 0.001	3
H1	Rosario	CO/NH4	1.5	0.36 ± 0.000	3
UBT1	Rosario	CO	1.5	0.36 ± 0.001	3
UBT1	Rosario	CO/NH4	1.5	0.36 ± 0.000	3
*A. vinelandii* WT	East Lansing	Sucrose	2.5	1.16 ± 0.009	2
*A. vinelandii nifD*-	Lansing	Sucrose/NH4	2.5	0.36 ± 0.000	2
*P. dioxanivorans*	Lansing	Glucose	2.5	0.36 ± 0.001	3
*P. dioxanivorans*	Lansing	Glucose/NH4	2.5	0.36 ± 0.000	3
H1	Lansing	H2/CO2	5	0.36 ± 0.002	2
H1	Lansing	H2/CO2/NH4	5	0.36 ± 0.000	3
UBT1	Lansing	H2/CO2	5	0.36 ± 0.000	2
UBT1	Lansing	H2/CO2/NH4	5	0.36 ± 0.000	3
P1-2	Lansing	H2/CO2	5	0.37 ± 0.001	2
P1-2	Lansing	H2/CO2/NH4	5	0.36 ± 0.000	3

## References

[b1] Canfield D. E., Glazer A. N. & Falkowski P. G. The evolution and future of Earth’s nitrogen cycle. Science 330, 192–6 (2010). 20929768 10.1126/science.1186120

[b2] Delwiche C. C. & Wijler J. Non-symbiotic nitrogen fixation in soil. Plant Soil 7, 113–129 (1956).

[b3] Franche C., Lindström K. & Elmerich C. Nitrogen-fixing bacteria associated with leguminous and non-leguminous plants. Plant Soil 321, 35–59 (2009).

[b4] Vitousek P. & Howarth R. Nitrogen limitation on land and in the sea: How can it occur? Biogeochemistry 13, 87–115 (1991).

[b5] Raymond J., Siefert J. L., Staples C. R. & Blankenship R. E. The natural history of nitrogen fixation. Mol. Biol. Evol. 21, 541–54 (2004). 14694078 10.1093/molbev/msh047

[b6] Schindelin H., Kisker C., Schlessman J. L., Howard J. B. & Rees D. C. Structure of ADP x AIF4(-)-stabilized nitrogenase complex and its implications for signal transduction. Nature 387, 370–6 (1997). 9163420 10.1038/387370a0

[b7] Rubio L. & Ludden P. Maturation of Nitrogenase : a Biochemical Puzzle. J. Bacteriol. 187, 405–14 (2005). 15629911 10.1128/JB.187.2.405-414.2005 PMC543557

[b8] Dos Santos P. C., Fang Z., Mason S. W., Setubal J. C. & Dixon R. Distribution of nitrogen fixation and nitrogenase-like sequences amongst microbial genomes. BMC Genomics 13, 162 (2012). 22554235 10.1186/1471-2164-13-162 PMC3464626

[b9] Dance I. Nitrogenase: a general hydrogenator of small molecules. Chem. Commun. 7, 10893–10907 (2013). 10.1039/c3cc46864j 24129752

[b10] Seefeldt L. C., Hoffman B. M. & Dean D. R. Mechanism of Mo-dependent nitrogenase. Annu. Rev. Biochem. 78, 701–22 (2009). 19489731 10.1146/annurev.biochem.78.070907.103812 PMC2814439

[b11] Rees D. C. *et al.* Structural basis of biological nitrogen fixation. Philos. Trans. A. Math. Phys. Eng. Sci. 363, 971–84; discussion 1035–40 (2005). 15901546 10.1098/rsta.2004.1539

[b12] Hwang J., Chen C. & Burris R. Inhibition of nitrogenase-catalyzed reductions. Biochimica et Biophysica Acta (BBA)- … 292, 256–270 (1973). 4705133 10.1016/0005-2728(73)90270-3

[b13] King G. M. & Crosby H. Impacts of plant roots on soil CO cycling and soil-atmosphere CO exchange. Glob. Chang. Biol. 8, 1085–1093 (2002).

[b14] Robson R. L. & Postgate J. R. Oxygen and hydrogen in biological nitrogen fixation. Annu. Rev. Microbiol. 34, 183–207 (1980). 6776883 10.1146/annurev.mi.34.100180.001151

[b15] Imlay J. A. Iron-sulphur clusters and the problem with oxygen. Mol. Microbiol. 59, 1073–1082 (2006). 16430685 10.1111/j.1365-2958.2006.05028.x

[b16] Hageman R. R. V. & Burris R. H. Nitrogenase and nitrogenase reductase associate and dissociate with each catalytic cycle. Proceedings of the National Academy of Sciences 75, 2699–702 (1978). 10.1073/pnas.75.6.2699 PMC392630 275837

[b17] Gallon J. R. & Wiley J. The oxygen sensitivity of nitrogenase : a problem for biochemists and micro-organisms Physi barrier s. Trends Biochem. Sci. 6, 19–23 (1981).

[b18] Zhao Y., Bian S.-M., Zhou H.-N. & Huang J.-F. Diversity of Nitrogenase Systems in Diazotrophs. J. Integr. Plant Biol. 48, 745–755 (2006).

[b19] Gadkari D., Morsdorf G., Meyer O. & Mörsdorf G. Chemolithoautotrophic assimilation of dinitrogen by *Streptomyces thermoautotrophicus* UBT1: identification of an unusual N2-fixing system. J. Bacteriol. 174, 6840–3 (1992). 1400234 10.1128/jb.174.21.6840-6843.1992 PMC207360

[b20] Gadkari D., Schricker K., Acker G., Kroppenstedt R. M. & Meyer O. *Streptomyces thermoautotrophicus* sp. nov., a Thermophilic CO- and H(2)-Oxidizing Obligate Chemolithoautotroph. Appl. Environ. Microbiol. 56, 3727–34 (1990). 16348374 10.1128/aem.56.12.3727-3734.1990 PMC185059

[b21] Ribbe M., Gadkari D. & Meyer O. N2 fixation by *Streptomyces thermoautotrophicus* involves a molybdenum-dinitrogenase and a manganese-superoxide oxidoreductase that couple N2 reduction to the oxidation of superoxide produced from O2 by a molybdenum-CO dehydrogenase. J. Biol. Chem. 272, 26627–33 (1997). 9334244 10.1074/jbc.272.42.26627

[b22] Hofmann-Findeklee C., Gadkari D. & Meyer O. In Catalysts for Nitrogen Fixation: Nitrogenases, Relevant Chemical Models and Commercial Processes (eds. Pedrosa F. O., Hungria M., Yates G. & Newton W. E.) 38, 23–30 (Springer, 2000).

[b23] Dietz A. & Mathews J. Classification of Streptomyces spore surfaces into five groups. Appl. Microbiol. 21, 527–33 (1971). 4928607 10.1128/am.21.3.527-533.1971 PMC377216

[b24] Frank J. A. *et al.* Critical evaluation of two primers commonly used for amplification of bacterial 16S rRNA genes. Appl. Environ. Microbiol. 74, 2461–70 (2008). 18296538 10.1128/AEM.02272-07 PMC2293150

[b25] Access O., Meier-Kolthoff J. P., Auch A. F., Klenk H.-P. & Göker M. Genome sequence-based species delimitation with confidence intervals and improved distance functions. BMC Bioinformatics 14, 60 (2013). 23432962 10.1186/1471-2105-14-60 PMC3665452

[b26] Kirby R. Chromosome diversity and similarity within the Actinomycetales. FEMS Microbiol. Lett. 319, 1–10 (2011). 21320158 10.1111/j.1574-6968.2011.02242.x

[b27] Acinas S. G., Marcelino L. A., Klepac-Ceraj V. & Polz M. F. Divergence and redundancy of 16S rRNA sequences in genomes with multiple rrn operons. J. Bacteriol. 186, 2629–35 (2004). 15090503 10.1128/JB.186.9.2629-2635.2004 PMC387781

[b28] Gao B. & Gupta R. S. Phylogenetic framework and molecular signatures for the main clades of the phylum Actinobacteria. Microbiol. Mol. Biol. Rev. 76, 66–112 (2012). 22390973 10.1128/MMBR.05011-11 PMC3294427

[b29] Verma M. *et al.* Phylogenetic analyses of phylum Actinobacteria based on whole genome sequences. Res. Microbiol. 164, 718–28 (2013). 23608518 10.1016/j.resmic.2013.04.002

[b30] Ouzounis C., Bork P. & Sander C. The modular structure of NifU proteins. Trends Biochem. Sci. 19, 1991–1992 (1994). 10.1016/0968-0004(94)90021-3 8048161

[b31] Meyer O., Frunzke K. & Morsdorf G. In Microbial Growth on C1 Compounds (eds. Murrell J. C. & Kelly D. P.) 433–59 (Intercept Ltd., 1993).

[b32] King G. M. & Weber C. F. Distribution, diversity and ecology of aerobic CO-oxidizing bacteria. Nat. Rev. Microbiol. 5, 107–18 (2007). 17224920 10.1038/nrmicro1595

[b33] Hofmann-Findeklee C. Molekularbiologische Untersuchung der Strukturgene des aeroben N2-fixierenden Systems von Streptomyces thermoautotrophicus sowie funktionelle Charakterisierung von rekombinantem SdnO. (Universität Bayreuth, 2000).

[b34] Grostern A. & Alvarez-Cohen L. Supplement-RubisCO-based CO2 fixation and C1 metabolism in the actinobacterium Pseudonocardia dioxanivorans CB1190. Environ. Microbiol. 2, 3040–3053 (2013). 23663433 10.1111/1462-2920.12144

[b35] Mahendra S. & Alvarez-Cohen L. Pseudonocardia dioxanivorans sp. nov., a novel actinomycete that grows on 1,4-dioxane. Int. J. Syst. Evol. Microbiol. 55, 593–8 (2005). 15774630 10.1099/ijs.0.63085-0

[b36] Dabundo R. *et al.* The Contamination of Commercial 15N2 Gas Stocks with 15N–Labeled Nitrate and Ammonium and Consequences for Nitrogen Fixation Measurements. PLoS One 9, e110335 (2014). 25329300 10.1371/journal.pone.0110335 PMC4201487

[b37] Wood P. M. The potential diagram for oxygen at pH 7. Biochem. J. 253, 287–289 (1988). 2844170 10.1042/bj2530287 PMC1149288

[b38] Hallenbeck P. C., Kostel P. J. & Benemann J. R. Purification and Properties of Nitrogenase from the Cyanobacterium, Anabaena cylindrica. Eur. J. Biochem. 98, 275–284 (1979). 111934 10.1111/j.1432-1033.1979.tb13186.x

[b39] Fawcett J. K. & Scott J. E. A Rapid and Precise Method for the Determination of Urea. J. Clin. Pathol. 13, 156–159 (1960). 13821779 10.1136/jcp.13.2.156 PMC480024

[b40] Gadkari D. Influence of the herbicides Goltix and Sencor on nitrification process in two soils. Zentralbl. Mikrobiol. 140, 547–554 (1985). 4090765

[b41] Kraut M., Hugendieck I., Herwig S. & Meyer O. Homology and distribution of CO dehydrogenase structural genes in carboxydotrophic bacteria. Arch. Microbiol. 152, 335–341 (1989). 2818128 10.1007/BF00425170

[b42] Hugendieck I. & Meyer O. The structural genes encoding CO dehydrogenase subunits (cox L, M and S) in Pseudomonas carboxydovorans OM5 reside on plasmid pHCG3 and are, with the exception of *Streptomyces thermoautotrophicus*, conserved in carboxydotrophic bacteria. Arch. Microbiol. 157, 301–304 (1992). 1510563 10.1007/BF00245166

[b43] Kruger B. & Meyer O. Thermophilic Bacilli growing with carbon monoxide. Arch. Microbiol. 139, 402–408 (1984).

[b44] Hurek T. *et al.* Occurrence of effective nitrogen-scavenging bacteria in the rhizosphere of kallar grass. Plant Soil 110, 339–348 (1988).

[b45] Yoshida N., Inaba S. & Takagi H. Utilization of atmospheric ammonia by an extremely oligotrophic bacterium, Rhodococcus erythropolis N9T-4. J. Biosci. Bioeng. 117, 28–32 (2014). 23849805 10.1016/j.jbiosc.2013.06.005

[b46] Hill S. & Postgate J. R. Failure of putative nitrogen-fixing bacteria to fix nitrogen. J. Gen. Microbiol. 58, 277–285 (1969). 5360480 10.1099/00221287-58-2-277

[b47] Lee S. *et al.* Molecular investigations on the dilemma of nitrogen-fixing characteristics ofAzotomonas. J. Basic Microbiol. 36, 99–105 (1996).

[b48] Montoya J. P., Voss M., Kahler P. & Capone D. G. A Simple, High-Precision, High-Sensitivity Tracer Assay for N (inf2) Fixation. Applied Environmental Micorbiology 62, 986–93 (1996). 10.1128/aem.62.3.986-993.1996 PMC1388808 16535283

[b49] Giller K. E. Use and abuse of the acetylene reduction assay for measurement of ‘associative’ nitrogen fixation. Soil Biol. Biochem. 19, 783–784 (1987).

[b50] Janda J. M. & Abbott S. L. 16S rRNA gene sequencing for bacterial identification in the diagnostic laboratory: pluses, perils, and pitfalls. J. Clin. Microbiol. 45, 2761–4 (2007). 17626177 10.1128/JCM.01228-07 PMC2045242

[b51] Meyer O. & Schlegel H. G. Biology of aerobic carbon monoxide-oxidizing bacteria. Annu. Rev. Microbiol. 37, 277–310 (1983). 6416144 10.1146/annurev.mi.37.100183.001425

[b52] Tobin-Janzen T. *et al.* Nitrogen changes and domain bacteria ribotype diversity in soils overlying the Centralia, Pennsylvania underground coal mine fire. SOIL Sci. 170, 191–201 (2005).

[b53] Schenk A. & Aragno M. Bacillus schlegelii, a new species of thermophilic, facultatively chemolithoautotrophic bacterium oxidizing molecular hydrogen. J. Gen. Microbiol. 115, 333–342 (1979).

[b54] Hamilton T. L. *et al.* Differential accumulation of nif structural gene mRNA in azotobacter vinelandii. J. Bacteriol. 193, 4534–4536 (2011). 21725008 10.1128/JB.05100-11 PMC3165501

[b55] Strandberg G. W. & Wilson P. W. Formation of the nitrogen-fixing enzyme system in Azotobacter vinelandii. Can. J. Microbiol. 14, 25–31 (1968). 5644401 10.1139/m68-005

[b56] Kämpfer P., Glaeser S. P. & Busse H.-J. Transfer of Bacillus schlegelii to a novel genus and proposal of Hydrogenibacillus schlegelii gen. nov., comb. nov. Int. J. Syst. Evol. Microbiol. 63, 1723–7 (2013). 22922537 10.1099/ijs.0.045146-0

[b57] Robinson A. C., Burgess B. K. & Dean D. R. Activity, reconstitution, and accumulation of nitrogenase components in Azotobacter vinelandii mutant strains containing defined deletions within the nitrogenase structural gene cluster. J. Bacteriol. 166, 180–186 (1986). 3457004 10.1128/jb.166.1.180-186.1986 PMC214574

[b58] Kieser T., Bibb M. J., Buttner M. J., Chater K. F. & Hopwood D. A. Practical streptomyces genetics. Published by the John Innes Foundation, ISBN 0-7084-0623-8, 613 pages (2000).

[b59] Margulies M. *et al.* Genome sequencing in microfabricated high-density picolitre reactors. Nature 437, 376–80 (2005). 16056220 10.1038/nature03959 PMC1464427

[b60] Bolger A. M., Lohse M. & Usadel B. Trimmomatic: a flexible trimmer for Illumina sequence data. Bioinformatics 30, 2114–2120 (2014). 24695404 10.1093/bioinformatics/btu170 PMC4103590

[b61] Bankevich A. *et al.* SPAdes: a new genome assembly algorithm and its applications to single-cell sequencing. J. Comput. Biol. 19, 455–77 (2012). 22506599 10.1089/cmb.2012.0021 PMC3342519

[b62] Liebert M. A., Zhang Z., Schwartz S., Wagner L. & Miller W. A Greedy Algorithm for Aligning DNA Sequences. 7, 203–214 (2000). 10.1089/10665270050081478 10890397

[b63] Consortium T. U. Activities at the Universal Protein Resource (UniProt). Nucleic Acids Res. 42, D191–8 (2014). 24253303 10.1093/nar/gkt1140 PMC3965022

[b64] Dunham J. P. & Friesen M. L. A cost-effective method for high-throughput construction of illumina sequencing libraries. Cold Spring Harb. Protoc. 2013, 820–34 (2013). 24003196 10.1101/pdb.prot074187 PMC3774519

[b65] Tritt A., Eisen J. a, Facciotti M. T. & Darling A. E. An integrated pipeline for de novo assembly of microbial genomes. PLoS One 7, e42304 (2012). 23028432 10.1371/journal.pone.0042304 PMC3441570

[b66] Green P. Documentation for PHRAP. Genome Center, University of Washington, Seattle. (1996) at www.phrap.org/phredphrap/phrap.html; date of access 21/06/2015.

[b67] Sievers F. *et al.* Fast, scalable generation of high-quality protein multiple sequence alignments using Clustal Omega. Mol. Syst. Biol. 7, 539 (2011). 21988835 10.1038/msb.2011.75 PMC3261699

[b68] Talavera G. & Castresana J. Improvement of phylogenies after removing divergent and ambiguously aligned blocks from protein sequence alignments. Syst. Biol. 56, 564–77 (2007). 17654362 10.1080/10635150701472164

[b69] Felsenstein J. PHYLIP-Phylogeny Inference Package. Cladistics 5, 164–166 (1989).

[b70] Tamura K., Stecher G., Peterson D., Filipski A. & Kumar S. MEGA6: Molecular Evolutionary Genetics Analysis version 6.0. Mol. Biol. Evol. 30, 2725–9 (2013). 24132122 10.1093/molbev/mst197 PMC3840312

[b71] Finn R. D. *et al.* Pfam: the protein families database. Nucleic Acids Res. 42, D222–30 (2014). 24288371 10.1093/nar/gkt1223 PMC3965110

