# Supplementary material for: Streptomyces thermoautotrophicus does not fix nitrogen
Source: Sci Rep. 2016 Feb 1;6:20086. doi: 10.1038/srep20086 (PMC4735515; doi:10.1038/srep20086)
Supplement: Supplementary Figures 1 to 3 [file srep20086-s1.pdf]

Title: *Streptomyces thermoautotrophicus* does not fix nitrogen

Authors:

Drew MacKellar[1,2], Lucas Lieber[3,4], Jeffrey S. Norman[5], Anthony Bolger[6], Cory Tobin[7], James W. Murray[4], Mehtap Oksaksin[6], Roger L. Chang[1], Tyler J. Ford[1], Peter Q. Nguyen[2], Jimmy Woodward[5], Hugo R. Permingeat[3], Neel S. Joshi[2], Pamela A. Silver[1,2], Björn Usadel[6], Alfred W. Rutherford[4], Maren L. Friesen[5], Jürgen Prell[6]

Supplementary Figure 1 - Strains H1 and P1-2 share 99% 16S rRNA sequence identity with strain UBT1. A multiple sequence alignment of one 16S rRNA gene from all 3 strains shows that the Sanger-sequenced fragment of 16S rRNA from strain P1-2 possesses 3 gaps and 1 mismatch relative to the corresponding H1/UBT1 gene sequence, over 1381bp of sequence; sharing 99.20% identity.

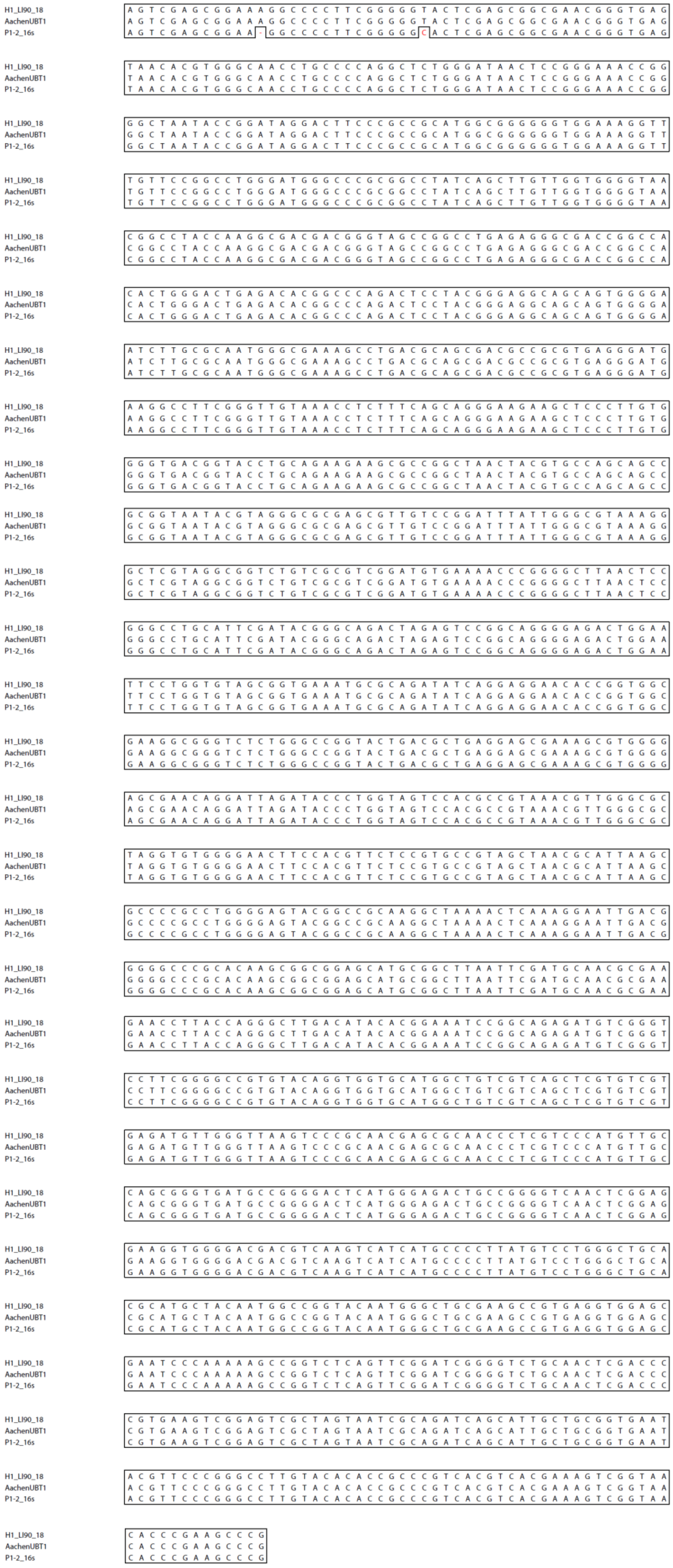

Supplementary Figure 2 - Consensus phylogenetic trees of actinomycetes with sequenced genomes based on small-subunit RNA genes (A) or concatenated ribosomal proteins (B). Clades containing the *S. thermoautotrophicus* branches and nearest neighbors are highlighted. Monophyletic clades outside of the nearest neighbor branches of *S. thermoautotrophicus* are collapsed up to the family level; other strains for which the entire species name is given are either incertae sedis, are the lone strain within their family for which data were available, or else failed to cluster with their family in this consensus.

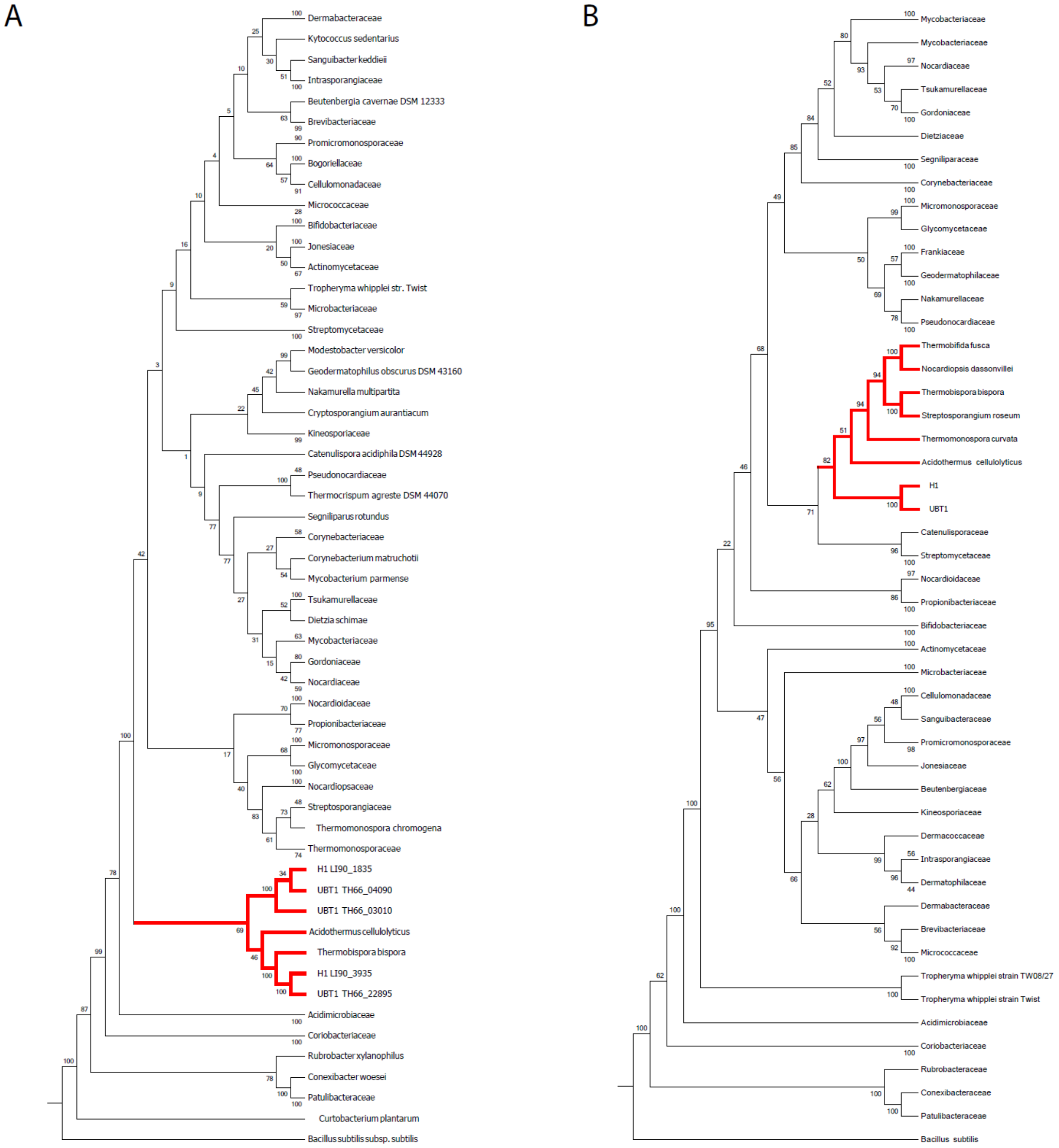

Supplementary Figure 3 - Contaminants present in commercial $^{15}N_2$ gas exceed any detected incorporation of label into biomass for strains H1 and UBT1. Values of contaminating $^{15}NH_4^+$ and $^{15}NO_3^-/NO_2^-$ recently reported[36] for the reagent used (Aldrich catalog #364584, Lot#SZ1670V) were used to calculate the amount of fixed ^{15}N that may have been present in the volume of gas introduced in experiments from the Boston site. Note that, as values for different lecture bottles from Lot#SZ1670V varied[36], the values showing the lowest concentration of fixed ^{15}N were used in the calculation.

$$n = PV/RT$$
$$P = 1atm$$
$$V = 27mL\ ^{15}N_2$$
$$R = 82.05736\frac{ml * ATM}{K * mol}$$
$$T = 298K$$

$$Average\ ^{15}N\ content\ detected\ in\ biomass\ samples:$$
$$H1:\ 0.034 \pm 0.006\mu M\ ^{15}N$$
$$UBT1:\ 0.016 + 0.008\mu M\ ^{15}N$$

$$Millimoles\ of\ ^{15}N_2\ added\ to\ the\ atmosphere\ in\ which\ samples\ were\ incubated:$$

$$\frac{1atm\ * 27mL\ ^{15}N_2}{82.05736\frac{mL * atm}{K * mol} * 298K} = \frac{27}{24,453.09}mol\ ^{15}N_2 = 0.0011mol\ ^{15}N_2$$

$$Potential\ contaminating\ ^{15}N(fixed)\ species\ present\ in\ gas\ prior\ to\ washing:$$
$$0.0011mol\ ^{15}N_2 * \frac{350\mu mol\ ^{15}NO_3/NO_2}{mol\ ^{15}N_2} \quad 0.385\mu mol\ of\ ^{15}N(fixed)$$

$$\frac{^{15}N(fixed)\ potentially\ present\ in\ unwashed\ gas}{Average\ ^{15}N\ content\ detected\ in\ biomass\ samples}:$$

$$H1:\ \frac{0.385\mu mol\ of\ ^{15}N(fixed)}{0.034\mu mol\ ^{15}N} = 11.3$$
$$UBT1:\ \frac{0.385\mu mol\ of\ ^{15}N(fixed)}{0.016\mu mol\ ^{15}N} = 24.1$$
